# Supplementary material for: Decline in coral cover and flattening of the reefs around Mauritius (1998–2010)
Source: PeerJ. 2018 Nov 29;6:e6014. doi: 10.7717/peerj.6014 (PMC6275115; doi:10.7717/peerj.6014)
Supplement: Table S2 — Long-term mean of local stressor by site for 1998–2010. Numbers in brackets indicate rank of each station. Rank goes from least to most abundant. See M&M for full site names. [file peerj-06-6014-s002.docx]

**Table S2.** Long-term mean of local stressor by site for 1998-2010. Numbers in brackets indicate rank of each station. Rank goes from least to most abundant. See M&M for full site names.

| **Site** | **Rainfall (mean volume in m^3^)** | **Agriculture (mean area in km^2^)** | **Population (mean # of people)** | **Tourism Index (mean # of tourists nights)** |
| --- | --- | --- | --- | --- |
| ALB | 9518194 (2) | 10.39 (1) | 80716 (6) | 26010 (3) |
| ALR | 35663887 (10) | 80.20 (10) | 120985 (8) | 2572868 (9) |
| BDT | 20660737 (6) | 49.78 (6) | 178310 (11) | 984656 (6) |
| BEN | 14986201 (4) | 21.78 (3) | 6217 (1) | 390639 (5) |
| BME | 35369531 (9) | 93.90 (11) | 103262 (7) | 1284260 (8) |
| BOM | 21343343 (7) | 40.40 (4) | 6422 (2) | 0 (1) |
| BVX | 6368169 (1) | 19.49 (2) | 10241 (3) | 0 (1) |
| PDO | 37276090 (12) | 116.14 (12) | 156769 (10) | 2820598 (11) |
| PSA | 11311215 (3) | 58.63 (7) | 186990 (12) | 347338 (4) |
| TBI | 36091907 (11) | 79.19 (9) | 121351 (9) | 2610749 (10) |
| TDO | 24796987 (8) | 60.12 (8) | 46207 (4) | 24492 (2) |
