# Supplementary material for: Decline in coral cover and flattening of the reefs around Mauritius (1998–2010)
Source: PeerJ. 2018 Nov 29;6:e6014. doi: 10.7717/peerj.6014 (PMC6275115; doi:10.7717/peerj.6014)
Supplement: Table S3 — Degree Heating Weeks data for the months of March to May 2005 showing that the coral reefs around Mauritius suffered severe thermal stress during this period. [file peerj-06-6014-s003.docx]

Table S3. Degree Heating Weeks (DHW) data from NOAA.

Source: https://coralreefwatch.noaa.gov/satellite/dhw.php

| (a) March 2005  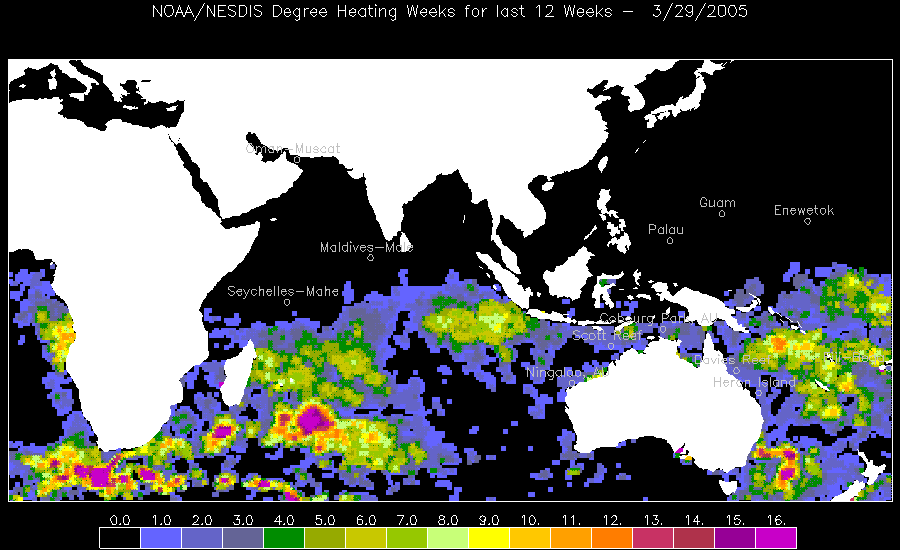 |
| --- |
| (b) April 2005  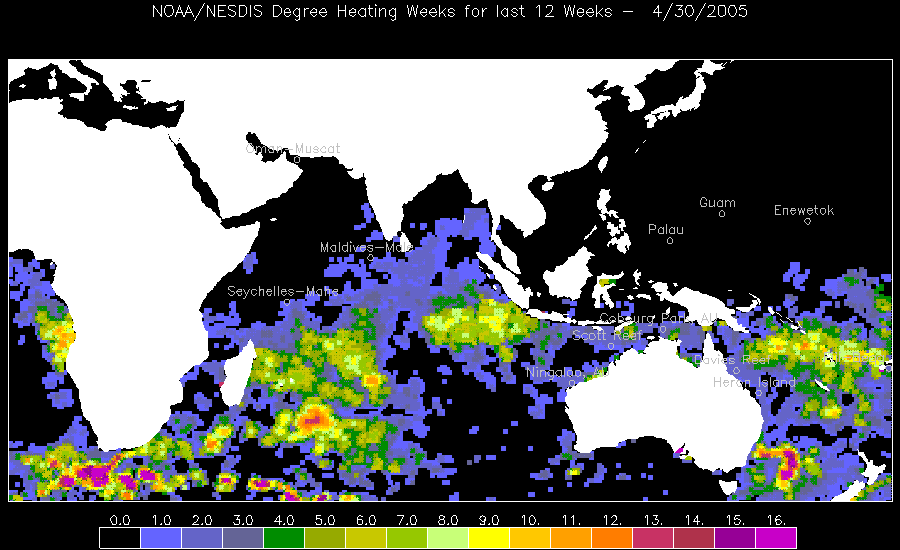 |
| (c) May 2005  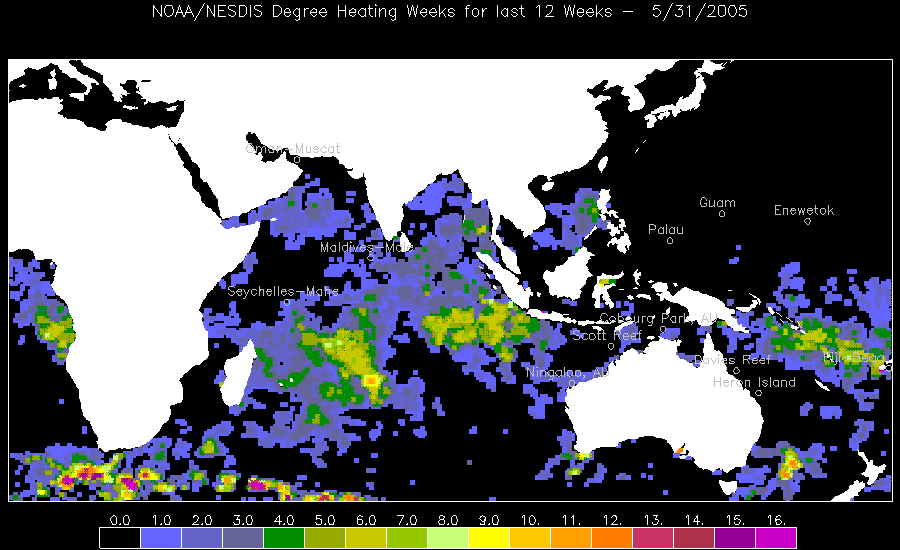 |
